# The Impact of Trophoblast Cell-Surface Antigen 2 Expression on the Survival of Patients with Gastrointestinal Tumors: A Systematic Review

**DOI:** 10.3390/jpm13101445

**Published:** 2023-09-28

**Authors:** Efstathia Liatsou, Dimitrios Schizas, Maximos Frountzas

**Affiliations:** 1 Department of Clinical Therapeutics, School of Medicine, Alexandra General Hospital, National and Kapodistrian University of Athens, 115 28 Athens, Greece; 2 First Department of Surgery, School of Medicine, Laikon General Hospital, National and Kapodistrian University of Athens, 115 27 Athens, Greece; 3 First Propaedeutic Department of Surgery, School of Medicine, Hippocration General Hospital, National and Kapodistrian University of Athens, 115 27 Athens, Greece

**Keywords:** TROP-2, gastrointestinal, tumor, surgery, survival

## Abstract

Background: Trophoblast cell-surface antigen 2 (TROP-2) is a transmembrane glycoprotein expressed in epithelial cells that has been associated with malignant progression in most carcinomas. Accordingly, the genetic complexity of gastrointestinal tumors necessitates the investigation of new biomarkers with potential prognostic value. The aim of this systematic review is to assess the effect of TROP-2 on the overall survival of patients who underwent surgery for gastrointestinal malignancy. Methods: The present systematic review was designed using the PRISMA and AMSTAR guidelines. We searched the Pubmed, EMBASE and CENTRAL databases from their inception to September 2023. Results: Ten studies that enrolled 2293 patients were included for qualitative analysis. Six studies evaluated patients with colorectal cancer, two studies included patients with gastric carcinoma, patients with pancreatic cancer were included in one study and one study included hepatobiliary cancer patients. TROP-2 was positive in 1005/2293 samples of the immunohistochemically evaluated biopsies and was associated with poor overall survival in all studies. High intensity was also associated with more aggressive histopathological characteristics, such us deep tissue invasion, lymph node metastasis and cell atypia. The prognostic value of TROP-2 was shown to be enhanced in patients with advanced disease and poor histological differentiation. Conclusion: TROP-2 was expressed at high levels in gastrointestinal tumors, which was associated with both tumor development and pathological aggressiveness. Therefore, TROP-2 could be used as a biomarker to determine clinical prognosis and as a potential therapeutic target in malignancies of the gastrointestinal tract, but further studies need to validate it.

## 1. Introduction

Trophoblast cell-surface antigen 2 (TROP-2), also known as tumor-associated calcium signal transducer 2 (TACSTD-2), is a cell-surface glycoprotein which acts as a calcium signal transducer and was first identified in the human placenta trophoblasts [1]. The functional role of TROP-2 has been thoroughly investigated at the molecular level with the cloning of the encoding gene and the identification of its post-translational structure [2]. The main actions of TROP-2 include activating the mitogen-activated protein kinase (MAPK) pathway as well as the extracellular signal-related kinase (ERK) and c-Jun N-terminal kinase (JKN) pathway that induce tumor cell survival, proliferation, invasion, migration and metastasis [2]. Its expression has been identified at high levels in various malignancies such as prostate, lung, breast and ovarian cancer, while normal somatic cells lack this signaling pathway activation [3]. Notably, in the case of gastrointestinal tract oncogenesis, the TROP-2 protein has been shown to be expressed at high levels in colon and gastric cancer and in hilar cholangiocarcinoma [4].

Although the expression and biological function of TROP-2 in primary tumors of the gastrointestinal tract has been investigated, the role of TROP-2 in patients’ prognosis and its related clinical implications as a prognostic biomarker still remain unclear. In a meta-analysis by Zeng et al. including 2569 patients, increased TROP-2 expression was associated with poor overall and disease-free survival outcomes across several solid tumors (HR: 1.896, 95% CI 1.599–2.247, *p* < 0.001) [5]. In addition, the expression of TROP-2 has been related to aggressive histopathological characteristics of different tumors, such us increased cell tumor growth, deep tumor depth and early vessel invasion [6]. On the other hand, a tumor-suppressive function has been reported for TROP-2 in cervical cancer, head and neck squamous-cell cancer and lung adenocarcinoma [7,8,9,10].

Such controversial findings demonstrate that further investigation is necessary to define the clinical role of TROP-2 as a tumor biomarker. The aim of the present systematic review is to clarify the prognostic value of TROP-2 in the survival of patients with gastrointestinal cancer and investigate its potential clinical implications based on data from the current literature.

## 2. Methods

### 2.1. Study Design

The recommendations of the Preferred Reporting Items for Systematic Reviews and Meta-Analyses (PRISMA) and AMSTAR-2 (A MeaSurement Tool to Assess systematic Reviews) were utilized for the design of the present systematic review [11,12].

### 2.2. Databases and Search

**Figure 1:**
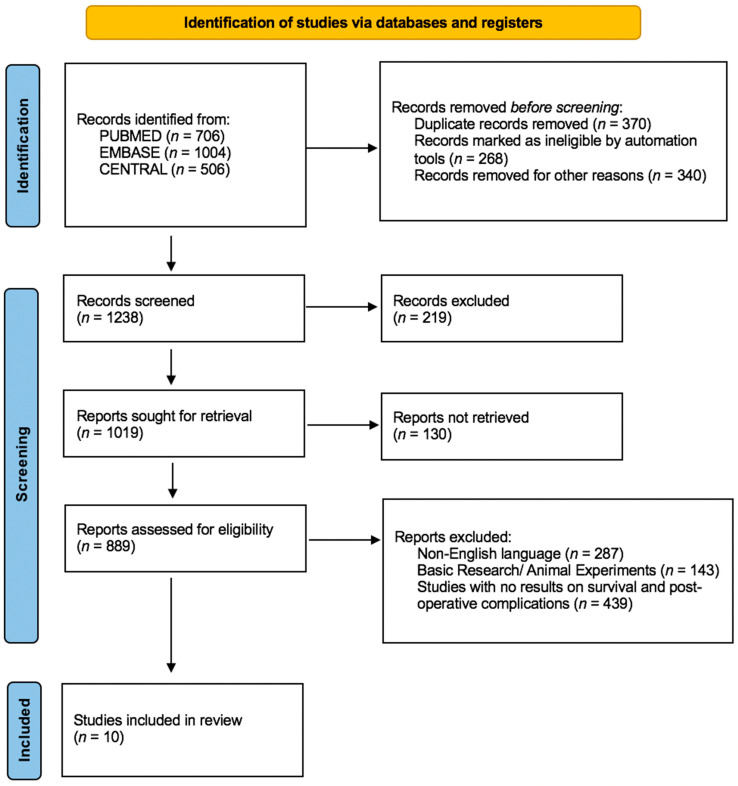
PRISMA 2020 flowchart of included studies.

The Medline, EMBASE and Cochrane Central Register of Controlled Trials CENTRAL databases were scanned from their inception to 1 September 2023. The main search algorithm was the same for the aforementioned databases and included the following terms (“trophoblast antigen 2, rat” [Supplementary Concept]) AND “Gastrointestinal Neoplasms” [Mesh]. The PRISMA flow diagram demonstrates the article selection process (Figure 1). Relevant articles were also found by scanning the references of the articles found (backward search) and locating newer articles that included the original cited papers (forward search).

Study selection was performed in three consecutive stages. First, duplicate publications were removed and then, the titles and abstracts of all electronic articles that appeared in the search were read in order to assess their eligibility. Second, full texts of all articles that met the inclusion criteria were downloaded and all observational studies were selected. Study search and data tabulation were conducted by two authors on similar predefined forms. The consensus of all authors resolved any possible conflicts after retrieving all available data.

### 2.3. Patients and Study Types

Authors predefined the eligibility criteria and no data restriction was taken into account during the search procedure. Studies were eligible for inclusion if they met the following criteria: (a) studies on adult patients (over 18 years of age); (b) studies included patients with gastrointestinal malignancies and measurement of trophoblast cell-surface antigen-2 (TROP-2) levels; (c) studies that reported oncological outcomes, especially overall survival, as a primary outcome. Finally, case reports, experimental animal studies, reviews and studies not written in English were excluded from this systematic review.

### 2.4. Study Outcomes

The outcomes that were investigated in the present systematic review were predetermined. Overall survival was the primary outcome, while secondary outcomes included several oncological parameters such as metastatic rate, cancer death, disease recurrence, disease-free survival and 5-year survival, where available.

### 2.5. Quality Assessment

**Figure 2:**
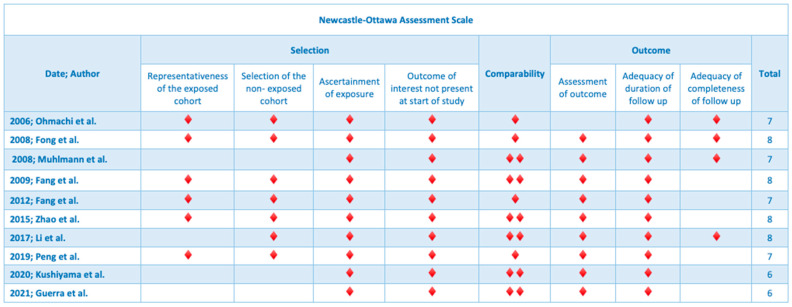
Methodological assessment of included studies using the Newcastle–Ottawa tool.

The Newcastle–Ottawa scale was utilized for the assessment of the methodological quality of the included articles [13]. Eight items, divided into three groups, were used for the assessment of the included studies: selection of study cohorts, comparability of cohorts and ascertainment of either the exposure or outcome of interest for case–control or cohort studies, respectively. After visual checking, studies were graded by stars for each item. Nine stars were the maximum award for each study (Figure 2).

### 2.6. Protocol Registration

Our systematic review was registered in the Open Science Framework (http://www.osf.io/ accessed on 9 September 2023) with the unique identifying number 10.17605/OSF.IO/YPTCH.

## 3. Results

A total of 10 studies that enrolled 22,931 patients met the predefined inclusion criteria and were included in the qualitative analysis of the present systematic review [14,15,16,17,18,19,20,21,22,23]. The search strategy is presented in the study flow diagram (Figure 1). All studies were retrospective cohort studies including patients who had undergone surgical resection due to gastrointestinal tumors. The median age of patients in the studies ranged from 58 to 72 years old and sex distribution was available in 10 studies, including 1311 males and 965 females. The primary outcome from all studies was overall survival, except Zhao et al., which considered lymph node metastasis as the primary outcome of the study [19]. Tissue specimens were formalin-fixed and paraffin-embedded, and anti-human TROP-2 antibodies were used for immunofluorescence.

Table 1 depicts the parameters of included studies and patients’ demographics. Table 2 demonstrates the tumor characteristics. In addition, antibody types, dilution protocols and histopathological scores that were utilized for immunohistochemistry are presented in Table 3. Table 4 describes the clinical outcomes of the included studies. Six studies examined TROP-2 expression in patients with colorectal cancer, two studies included patients who had undergone surgery for gastric carcinoma, one study evaluated TROP-2 expression in patients with pancreatic cancer and one study analyzed tissue specimens from gallbladder carcinomas.

### 3.1. Colorectal Cancer

A total of 1147 patients from six studies were evaluated regarding the clinical significance of TROP-2 expression from colon cancer tissue biopsies. First, Ohmachi et al. from Japan performed a cDNA microarray analysis of genes that were associated with oncogenesis in colorectal cancer [15]. Higher expression was observed particularly in tumor cells (*p* < 0.01), according to mRNA expression, which was also found to be higher in comparison with normal cells [15]. In addition, high TROP-2 expression was associated with higher incidence of liver metastasis (34.6% vs. 8.3%, *p* = 0.005) and cancer death (34.6% vs. 14.6%, *p* = 0.064) [15]. The Cox proportional hazard model correlated TROP-2 status with overall survival in univariable (RR = 2.03, 95% CI 1.22–3.50, *p* = 0.007) and multivariable analysis (RR = 2.38 95% CI 1.29–4.74, *p* = 0.005) [15].

Similarly, Fang et al. from the Department of Colorectal Surgery in the State Key Laboratory of Oncology in Southern China retrospectively evaluated the impact of the cell-surface receptor TROP-2 in 620 patients who had undergone surgery for colon cancer [17]. Additive chemotherapy had been implemented in 311 patients intraoperatively and in 533 patients after operation [17]. Histological and clinical data were collected in a follow-up period of 52 months and the plotted Kaplan–Meier survival curves showed that higher levels of TROP-2 were related to poorer prognosis and earlier disease recurrence (5-year survival, HR = 1.43, 95% CI 1.04–1.97, *p* = 0.03 and disease recurrence HR = 1.38, 95% CI 1.01–1.89, *p* = 0.043) [17]. The same study group focused on 220 patients with Stage IIb colorectal cancer and TROP-2 expression levels were found to be a possible important prognostic factor for survival (5-year survival, HR = 0.959, 95% CI 0.897–1.026, *p* = 0.223), but statistical significance was not reached [18].

In the Department of Surgery of Hebei Medical University in China, immunofluorescence analysis of TROP-2 was performed in paraffin-embedded colon cancer tissue specimens of 82 patients who had undergone surgery for colon cancer and had been treatment-naïve pre-operatively [19]. Histopathological results outlined that the expression of TROP-2 was linked with lymph node metastasis and Dukes Stage C + D (*p* = 0.018 and *p* = 0.02, respectively) [19]. A Chinese retrospective study by Peng et al. evaluated patients with a histologically diagnosed colorectal adenocarcinoma with liver metastasis who had undergone surgery and metastasectomy with a mean follow-up of 35 months [21]. TROP-2 expression was found to be high in patients with a poor histopathological grade (35.9% vs. 15.4%, *p* = 0.007) and higher preoperative CA 19-9 levels (67.2% vs. 41.5%, *p* = 0.003) [21]. TROP-2 was found to be an important prognostic factor in terms of recurrence-free survival in univariable (HR = 2.017, 95% CI 1.198–3.396, *p* = 0.008) and multivariable analysis (HR = 1.877, 95% CI 1.091–3.230, *p* = 0.023), as well as overall survival (HR = 2.090, 95% CI (1.037–4.214), *p* = 0.039) [21].

In Guerra et al., high expression of TROP-2 was found in 40 of 80 patients and was associated with lymph node metastasis (N0: 40% vs. N1–N2: 60%, *p* = 0.047) [23]. In the same study, high levels of TROP-2 (threshold of 88%) were associated with higher death rates (57.1% vs. 37.8%, *p* = 0.020) in a subgroup of 53 cases.

### 3.2. Gastric Cancer

TROP-2 expression in patients with gastric cancer along with their clinical outcomes was evaluated in two studies. In 2008, Muhlmann et al. from Austria determined the expression of TROP-2 in 47 patients who had undergone gastrectomy and 57 patients who had undergone subtotal gastrectomy [16]. Diffuse-type gastric cancer was correlated with significantly higher expression of TROP-2, which was also associated with deeper wall invasion (*p* = 0.02), whereas in patients with intestinal-type gastric carcinoma, high levels of TROP-2 were correlated with shorter disease-free survival (*p* = 0.03) [16]. Kaplan–Meier curves demonstrated significantly poorer disease-free and overall survival (*p* < 0.01 and *p* = 0.01, respectively) both in patients with advanced intestinal-type gastric cancer and patients with lymph node metastasis [16]. Multivariable analysis showed that the transmembrane glycoprotein was a significant prognostic factor for patients with intestinal-type gastric carcinoma in terms of disease-free survival (RR = 6.39, 95% CI 2.20–18.55, *p* < 0.01) [16].

This was also confirmed by Kushiyama et al., who presented the largest sample size (740 patients) among the included studies [22]. Higher expression of TROP-2 was associated with the clinicopathological characteristics of poor histological differentiation (58.18% vs. 37.56%, *p* < 0.0001), T3–T4 tumor depth (40% vs. 53.38%, *p* = 0.0003), lymph node metastasis (57.43% vs. 36.97%, *p* < 0.0001) and lymphatic (54.8% vs. 34.42%, *p* < 0.0001) and venous invasion (68.57% vs. 41.49%, *p* < 0.0001) [22]. Univariable analysis showed that poor overall survival was significantly correlated with TROP-2 overexpression (HR = 1.562, 95% CI 1.221–1.998, *p* < 0.01), but it was not confirmed in the multivariable analysis [22].

### 3.3. Pancreatic and Hepatobiliary Cancer

One study by Fong et al. highlighted that patients with pancreatic cancer who had positive TROP-2 staining presented poorer differentiation, lymph node metastasis and aggressive histopathological stage [14]. TROP-2 overexpression along with a negative resection margin were found to be an independent prognostic marker in terms of overall survival (RR = 1.8, 95% CI 1.1–3.1, *p* = 0.01) [14]. Concerning progression-free survival, TROP-2 overexpression was independent of nodal status and margin involvement by the tumor (RR = 1.8, 95% CI 1.1–2.9, *p* = 0.014) [14].

Regarding hepatobiliary cancer, Li et al. included 105 patients with confirmed gallbladder cancer who had undergone resection of primary tumor in their study [20]. Interestingly, worrisome clinicopathological features, such as poor histological grade (well or moderate: 38.8% vs. poor: 86.3%, *p* = 0.000), aggressive tumor invasion (T1–T2: 44.4% vs. T3–T4: 80.4%, *p* = 0.000), lymph node metastasis (N0: 43.1% vs. N1–2:85.1%, *p* = 0.001) and higher TNM stage (I–II: 23.7% vs. III–IV: 83.6%, *p* = 0.000) were strongly associated with high TROP-2 expression [23]. Cox regression analysis confirmed that TROP-2 expression and tumor invasion were two independent prognostic factors for gallbladder cancer in univariable (RR = 0.259, 95% CI 0.163–0.412, *p* = 0.0000) and multivariable analysis (RR = 0.463, 95% CI 0.274–0.782, *p* = 0.004) [20].

## 4. Discussion

The present systematic review depicts the association of TROP-2 with important histopathological characteristics, such as depth of epithelial wall invasion and lymphatic or vascular invasion, as well as clinical outcomes including overall survival and progression-free survival in patients with gastrointestinal tumors that have undergone surgical operation. Findings remained consistent regarding all tumors of the gastrointestinal tract. First, patients with colorectal cancer presented higher levels of TROP-2 in tumor cells compared to normal tissue cells. Higher TROP-2 expression was also associated with aggressive histopathological stage, early lymph node invasion and tumor metastasis. Concerning survival, disease recurrence was more common in patients with highly expressed TROP-2 tumors. That was also an independent prognostic factor for overall survival and recurrence-free survival in case of existing metastatic disease. This finding was remarkably unanimous among all studies and statistical significance depended mainly on the sample size of the study. Similarly, in gastric cancer, the impact of TROP-2 expression on the clinicopathological features of the tumor depended on the tumor histological subtype. For example, in diffuse-type gastric cancer, cells with high TROP-2 expression were more often stage T3–T4, whereas the intestinal-type was associated with poorer prognosis. Moreover, TROP-2 expression played a prognostic role in patients with advanced disease and lymph node metastasis. Lastly, the prognostic role of TROP-2 was underlined in the case of pancreatic and hepatobiliary cancer, as a strong association between aggressive cancer behavior and short life span was demonstrated.

TROP-2 has been thoroughly examined as a potential biomarker for early diagnosis, but also a prognostic factor and potential therapeutic target for several solid malignancies [24]. First, the prognostic role of TROP-2 has been examined in different histological types of lung cancer. In a study by Inamura et al., high TROP-2 expression levels were associated with high mortality in patients with adenocarcinoma (overall survival, HR = 1.49, 95% CI 1.06–2.13, *p* = 0.021) and longer overall survival in patients with high-grade neuroendocrine tumor (HGNET), (HR = 0.50, 95% CI 0.12–0.65, *p* = 0.0015), whereas no association was found with squamous-cell carcinoma (HR = 1.33, 95% CI 0.74–25.8), *p* = 0.35) [25]. Findings from Italiano et al. were unanimous in advanced non-small-cell lung cancer (NSCLC), as shorter median overall survival for patients was demonstrated (12.6 vs. 16.3 months, *p* = 0.007) [26]. In early triple negative breast cancer, patients with high TROP-2 had higher rates of nodal involvement (53%) compared to patients with medium (23%) and low (21%) TROP-2 expression (*p* = 0.03) and more frequent lymphovascular invasion (65% of patients with high TROP-2 vs. 15–16% of patients with medium and low TROP-2 expression, *p* ≤ 0.001) [27]. Amborgi et al. showed that TROP-2 is an unfavorable prognostic factor when localized in the membrane of breast cancer cells in terms of overall survival (HR = 1.63, *p* = 0.04), whereas a favorable effect was observed in intracellular expression (HR = 0.48, *p* = 0.003) [28]. In epithelial ovarian cancer, a series of 128 paraffin-embedded biopsies showed that patients with high levels of TROP-2 had shorter median overall survival (52 vs. 20 months, *p* < 0.01) [29].

The current systematic review is the first in the literature which summarizes the impact of TROP-2 expression on the survival of patients with resected gastrointestinal tumors. An additional strength of this systematic review is the quality evaluation of the selected articles, as well as the study registration in an international database (OSF). The quality assessment performed according to the Newcastle–Ottawa tool indicated high methodological quality (seven to eight stars) for the majority (eight) of the included studies, which improves the methodological quality of the present systematic review. Moreover, the search and selection of the articles were done by two independent researchers to limit selection bias.

However, our study presents some limitations that need to be stated. First, there is no standardized method for the immunohistochemical assessment of TROP-2 expression in tumors, a fact that may affect the reproducibility and generalizability of the results. Considerable heterogeneity in the type of TROP-2 antibody and its dilution grade, the protocol of paraffin elaboration and immunohistochemistry analysis was noticed among studies. In addition, a quantitative interpretation of study outcomes would not be possible, as differences were observed in the evaluation scores, the cut-off for high TROP-2 expression as well as the pathologists who examined the final tissues, thus performance bias may have been introduced (Table 3). Therefore, although overall survival was the primary outcome in the majority of studies, a pooled meta-analysis was not feasible. Moreover, the retrospective methodological strategy of the included studies, as well as the time interval (2006–2022) of study publication concerning the evolution in surgical techniques throughout this timeline might have induced a possible lead-time bias in our study. Lastly, the limited and single-centered examined populations raise cautiousness regarding the value of clinical evidence; thus, these findings need to be validated in multicenter studies or larger cohort studies.

In the current area of precise oncology, a growing body of evidence suggests the role of TROP-2 as a promising molecular target for the treatment of various malignancies, along with the simultaneous development of antibody-drug conjugates (ADCs). The efficacy of sacituzumab govitecan (IMMU-132), which contains SN-38, the active metabolite of irinotecan, was compared with chemotherapy in 468 patients with relapsed triple negative breast cancer in the ASCENT III trial, which achieved its primary endpoint with a median overall survival of 12.1 months with sacituzumab govitecan vs. 6.7 months with chemotherapy (HR = 0.48, *p* < 0.001) [30]. The following biomarker analysis of the study showed that regardless of TROP-2 expression, patients with metastatic TNBC benefited from sacituzumab govitecan in comparison to physician’s choice chemotherapy; thus, TROP-2 expression is not currently recommended as a biomarker predicting the benefits of sacituzumab govitecan [31]. A large number of ongoing studies with new agents have been designed and recruiting patients, but most studies are phase I/II basket trials that include patients with TNBC, HR+ breast cancer, SCLC, NSCLC, urothelial cancer and colorectal cancer [32]. However, the efficacy outcomes of such agents have not yet been presented in tumors of the gastrointestinal tract, and may potentially be derived only in the context of basket trials (NCT05489211), raising the concern of little direct comparison with other therapeutic agents to extract concrete data [33]. This systematic review highlights the prognostic role of TROP-2 as a biomarker for gastrointestinal tumors and emphasizes the need for the further validation of the conclusions derived from in vitro retrospective clinical studies to a more prospective and randomized in vivo setting in order to elucidate its therapeutic possibilities.

## Figures and Tables

**Table 1:** Characteristics of included studies.

Year; Author	Type of Study	Center/Country	Number of Patients	Age (Mean, Range)	Gender
2006; Ohmachi et al.	Retrospective	Department of Surgery and Molecular Oncology, Medical Institute of Bioregulation, Kyushu University	N = 16	67 (33–84)	Males: 44 Female: 30
2008; Fong et al.	Retrospective	Department of Hematology and Oncology/Austria	N = 197	67 (37–91)	Males: 111 Female: 86
2008; Muhlmann et al.	Retrospective	Center of Operative Medicine, Department of Visceral, Transplant and Thoracic Surgery, Innsbruck Medical University	N = 104	70 (30–94)	Males: 70 Female: 34
2009; Fang et al.	Retrospective	Department of Colorectal Surgery, State Key Laboratory of Oncology in Southern China	N = 620	59 (15–86)	Males: 364 Female: 256
2012; Fang et al.	Retrospective	Department of Colorectal Surgery, State Key Laboratory of Oncology in South China, Sun Yat-sen University Cancer Center	N = 220	59 (7–86)	Male: 130 Female: 90
2015; Zhao et al.	Retrospective	Department of Surgery, Hebei Medical University	N = 82	59.8 (35–90)	Male: 53 Female: 30
2017; Li et al.	Retrospective	Department of General Surgery, Changzheng Hospital, The Second Military Medical University	N = 105	NA	Male:30 Female: 75
2019; Peng et al.	Retrospective	Department of Colorectal Surgery, Department of Experimental Research/China	N = 129	58 (25–78)	Males: 78 Female:51
2020; Kushiyama et al.	Retrospective	Departments of Gastroenterological Surgery, and Molecular Oncology and Therapeutics	N = 740	ΝA	Males: 431 Female: 309
2021; Guerra et al.	Retrospective	Laboratory of Cancer Pathology, Center for Advanced Studies and Technology (CAST)	N = 80	72 (30–88)	Males: NA Female: NA

**Table 2:** Characteristics of tumors of enrolled patients.

Year; Author	Type of Cancer	Stage	Surgery	Margins	Metastasis	Systematic Therapy
2006; Ohmachi et al.	Colorectal cancer	NA	Yes: 34	NA	Yes: 13 No: 61	NA
2008; Fong et al.	Pancreatic ductal adenocarcinoma	Ia: 6 Ib: 24 IIa: 34 IIb: 70 III: 20 IV: 26	Yes: 143 No: 54	R0: 86 R1: 42	Yes: 25 No: 117	NA
2008; Muhlmann et al.	Gastric cancer	Ia: 15 Ib: 22 II: 30 III:19 IV: 18	Yes: 104 Total gastrectomy: 47 Subtotal gastrectomy: 57	R0: 97 R1: 7	Yes:10 No: 94	Neoadjuvant: 6
2009; Fang et al.	Colorectal cancer	I: 86 IIa: 146 IIb: 110 IIIa: 10 IIIb: 106 IIIc: 24 IV: 124	Yes: 620	NA	Yes: 225 No: 395	Neoadjuvant: 311 Adjuvant: 533
2012; Fang et al.	Colorectal cancer	IIb: 220	Yes: 220	NA	NA	NA
2015; Zhao et al.	Colorectal cancer	NA	Yes: 82	NA	NA	0
2017; Li et al.	Gallbladder cancer	I–II: 38 III–IV: 67	Yes: 105	NA	NA	NA
2019; Peng et al.	Colorectal primary tumor and liver metastasis	NA	Yes: 129	NA	Yes: 129	Neoadjuvant: 31 Adjuvant: 87 Radiation: 5
2020; Kushiyama et al.	Gastric carcinoma	NA	Yes: 740	NA	Yes: 30/No: 710	0
2021; Guerra et al.	Colorectal cancer metastasis	NA	Yes: 80	NA	Yes: 43/No: 37	NA

**Table 3:** Methodological features of included studies.

Year; Author	Type of Biopsy	Type of TROP-2 Antibody	TROP-2 Score	Follow-Up (Median)	Primary Outcome
2006; Ohmachi et al.	Formalin-fixed, paraffin-embedded	Purified recombinant human TROP-2 extracellular domain (R&D Systems, Inc., Minneapolis, MN, USA) (5 Ag/mL)	NA	NA	Overall survival
2008; Fong et al.	Formalin-fixed, paraffin-embedded	Purified goat polyclonal antibody detecting the recombinant human TROP-2 extracellular domain at a dilution of 1:50	Proportion: 0: none, 1: <10%, 2: 10–50%, 3: 51–80%, 4: >80%)	NA	Overall survival
			Intensity: 0, 1: no staining, 2: weak, 3: moderate, 4: strong)		
2008; Muhlmann et al.	Formalin-fixed, paraffin-embedded	Recombinant human TROP-2 extracellular domain at a dilution of 1:50 (AF650, R&D Systems, Minneapolis, MN, USA)	Proportion: 0: none, 1: 10%; 2: 10–50%; 3: 51–80%; 4: 80%	NA	Overall survival
			Intensity: 0: no staining, 1: weak, 2: moderate, 3: strong		
2009; Fang et al.	Formalin-fixed, paraffin-embedded	Dilution of antibody for staining was 1:10 for TROP-2 (monoclonal goat, R&D Systems, Inc.)	Proportion: 0: none, 1: <10%, 2: 10–50%, 3: >50%	52 months (range 1–130 months)	Overall survival
			Intensity: 0: none, 1: weak, 2: moderate, 3: strong		
2012; Fang et al.	Formalin-fixed, paraffin-embedded	Dilution of antibody for staining was 1:50 for TROP-2 (monoclonal goat, R&D systems, Inc.)	Proportion: 0: none, 1: <10%, 2: 10–50%, 3: >50%	103 months (range, 1–167 months)	Overall survival
			Intensity: 0: negative, 1: weak, 2: moderate, 3: positive		
2015; Zhao et al.	Formalin-fixed, paraffin-embedded	Rabbit anti-human TROP-2 polyclonal antibody (1:50; Santa Cruz Biotechnology, Inc., Dallas, TX, USA)	NA	NA	Lymph node metastasis
2017; Li et al.	Formalin-fixed, paraffin-embedded	NA	NA	NA	Overall survival
2019; Peng et al.	Formalin-fixed, paraffin-embedded	A primary TROP-2 antibody (1:500 dilution, ab214488; Abcam, Cambridge, UK)	Proportion: 0: <5%, 1: 5–24%, 2: 25–49%, 3: 50–74%, 4: 75–100%	35 months (2–143)	Overall survival
			Intensity: 0: negative, 1: weak, 2: moderate, 3: strong		
2020; Kushiyama et al.	Formalin-fixed, paraffin-embedded	Anti-mouse antibody for TROP-2 (1:250, sc-376746, Santa Cruz Biotechnology)	Proportion: 0: 0%, 1: 1–30%, 2: 31–70%, 3: 71–100%	NA	Overall survival
			Intensity: 0, 0: 1, weak: 2: moderate, 3: strong		
2021; Guerra et al.	Formalin-fixed, paraffin-embedded	Anti-goat (sc-2020; Santa Cruz Biotechnology) pAbs	NA	400 months	Overall survival

**Table 4:** Outcomes of included studies. RR = relative risk.

Year; Author	TROP-2 Expression		Overall Survival (Median, Range)		Univariate Analysis	Multivariate Analysis
			TROP-2 (−)	TROP-2 (+)	RR (95% C.I.)	RR (95% C.I.)
2006; Ohmachi et al.	Yes: 34 Strong: 7 Moderate: 9 Weak: 18	No: 0	NA	NA	2.02 (95% 1.22–3.50), *p* = 0.007	2.38 (95% 1.29–4.74), *p* = 0.005
2008; Fong et al.	Yes: 109 Strong: 57 Moderate: 52 Low: 76 0: 12	No: 88	14 months	8 months (*p* < 0.01)	NA	1.8 (95% 1.1–3.1), *p* < 0.01
2008; Muhlmann et al.	Yes: 58	No: 46	52 months (range 1–163): intestinal-type carcinoma 16 months (range 1–54): diffuse-type carcinoma		*p* = 0.97	6.3 (2.2 to 18.5)
2009; Fang et al.	Yes: 155	No: 465	NA	NA	1.43 (95% 1.04–1.97), *p* = 0.03	1.14 (95% 0.55–1.38)
2012; Fang et al.	NA	NA	NA	NA	0.959 (95% 0.897–1.026)	NA
2015; Zhao et al.	Yes: 75	No: 7	NA	NA	NA	NA
2017; Li et al.	Yes: 65	No: 40	NA	NA	0.259 (0.163–0.412), *p* = 0.000	0.463 (0.274–0.782), *p* = 0.004
2019; Peng et al.	Yes: metastasis 65/129 primary tumor 49/70	No: metastasis 64/129 primary tumor 21/70	NA	NA	2.090 (95% 1.037–4.214)	2.090 (95% 1.037–4.214), *p* < 0.039
2020; Kushiyama et al.	Yes: 330 Strong: 23 Moderate: 35 Moderate to strong: 58	No: 410	NA	NA	1.562 (1.221–1.998), *p* = 0.0004	1.249 (0.959–1.628), *p* = 0.0994
2021; Guerra et al.	Yes: 65 High: 40	No: 15	NA	NA	1.96, *p* = 0.00058	NA

## Data Availability

All data are available upon reasonable request.
